# Integrating Nano-Cu_2_O@ZrP into In Situ Polymerized Polyethylene Terephthalate (PET) Fibers with Enhanced Mechanical Properties and Antibacterial Activities

**DOI:** 10.3390/polym11010113

**Published:** 2019-01-10

**Authors:** Jialiang Zhou, Xiang Fei, Congqi Li, Senlong Yu, Zexu Hu, Hengxue Xiang, Bin Sun, Meifang Zhu

**Affiliations:** State Key Laboratory for Modification of Chemical Fibers and Polymer Materials, International Joint Laboratory for Advanced Fiber and Low-dimension Materials, College of Materials Science and Engineering, Donghua University, Shanghai 201620, China; zjl19871217@126.com (J.Z.); xiangfei@dhu.edu.cn (X.F.); licq1006@163.com (C.L.); 18317129897@163.com (S.Y.); wind007007@hotmail.com (Z.H.); sunbin@dhu.edu.cn (B.S.)

**Keywords:** nano-Cu_2_O@ZrP, PET, antibacterial fibers, in situ polymerization

## Abstract

The approach of in situ polymerization modification has proven to be an effective route for introducing functions for polyester materials. In this work, Cu_2_O@ZrP nanosheets with excellent dispersity and high antibacterial activity were integrated into in situ polymerized polyethylene terephthalate (PET) fibers, revealing an enhanced mechanical performance in comparison with the PET fibers fabricated directly via a traditional melt blending method. Additionally, such an in situ polymerized PET/Cu_2_O@ZrP fibers displayed highly enhanced mechanical properties; and great antibacterial activities against multi-types of bacterium, including *S. aureus*, *E. coli* and *C. albicans*. For the as-obtained two types of PET/Cu_2_O@ZrP fibers, we have detailed their molecular weight (detailed molecular weight) and dispersibility of nano-Cu_2_O@ZrP and fibers crystallinity was investigated by Gel chromatography (GPC), Scanning electron microscope (SEM), and X-ray diffractometer (XRD), respectively. The results showed that the aggregation of the nano-Cu_2_O@ZrP in the resultant PET matrix could be effectively prevented during its in situ polymerization process, hence we attribute its highly enhanced mechanical properties to its superior dispersion of nano-Cu_2_O@ZrP.

## 1. Introduction

As one of the most widely used low-cost polymers, poly(ethylene terephthalate) (PET) materials featured with excellent thermal and chemical stability and mechanical performance [1,2,3], have been widely applied to various areas, such as textile fibers, films and engineering plastics [4,5,6]. In recent years, great attention on PET or other polyester fabrics has shifted from single comfort to a health management function, among which antibacterial function is considered to be a critical indicator. However, it is well known that neat PET materials have non-antibacterial properties, instead porous structures in PET fabrics brought by weaving is beneficial to the adhesion, growth and proliferation of bacteria [7]. As a result, current strategies of endowing PET-based fabrics antibacterial functions are mainly focused on introducing various antibacterial active nanomaterials (e.g., Ag and Ag-based compounds, TiO_2_, Mg(OH)_2_ and ZnO NPs, etc.) [8,9,10,11,12]. To realize the aforementioned antibacterial hybrid fabrics based on PET, three main methods have been widely implemented, i.e., surficial coating, melting blending, and in situ polymerization [13,14,15]. Among them, coating antibacterial-active nanomaterials onto the PET fabric surfaces is always facing the drawback of laundering durability due to a lack of adhesion forces. Alternatively, the melt blending-spinning technique with merits of high efficiency and low-cost is applied to solve the problem of laundering durability [16]. For example, through a melting-and-mixing technology, Yimin Zhu et al. reported a kind of antibacterial PET master-batch by using nano-Mg(OH)_2_ (size: ~500–1300 nm) as the antibacterial fillers [13,17]. Such an antibacterial PET/nano-Mg(OH)_2_ master-batch (containing 5 wt% Mg(OH)_2_) can still possess a high antibacterial rate of more than 90% even after 50 times of washing. Even so, many other problems (e.g., high additive amount, nanoparticle aggregation and interface compatibility with matrix) still exist in the melt blending-spinning technique; and have limited its further application [14]. As a result, great attention is therefore triggered onto the in situ polymerization technique, which is highly expected to overcome the aforementioned drawbacks in producing PET-based functional materials. Unfortunately, so far, only a few works have been demonstrated regarding the in situ polymerization method for the fabrication of antibacterial PET composite material [18,19]. Accordingly, further exploration of the in situ polymerization method is highly desired, which will serve as the foundation for developing a new kind of antibacterial PET material for a wide variety of application-oriented fields in the future.

Recently, Cu_2_O and Cu_2_O-based nanomaterials have attracted great attention as effective and broad-spectrum antibacterial agents; and with its low cost and high safety, they have been practically used in various antibacterial fields [20,21,22,23,24,25]. However, the strategy of applying such Cu_2_O or Cu_2_O-based nanomaterials to antibacterial fibers has yet to be proposed and demonstrated. 

Herein, considering the small size of Cu_2_O as well as its aggregation effect, a kind of Cu_2_O@ZrP micro-nano composite by loading Cu_2_O onto ZrP nanoflakes was first fabricated. Afterwards, through an in situ polymerization method, Cu_2_O@ZrP composite could be successfully and uniformly integrated into PET fibers, presenting highly enhanced mechanical properties and antibacterial activities when compared to its control sample obtained by the melt-blending method. In addition, the dispersity of nano-Cu_2_O@ZrP in the corresponding PET matrix and crystalline change of PET polymers fabricated were also compared and discussed in detail. We highly expect that in situ polymerized PET/Cu_2_O@ZrP hybrid fibers will gain huge marketing potential in high-end biomedical textiles, protective clothing and other fields in the future. 

## 2. Experimental Section

### 2.1. Materials

α-ZrP (~500–1200 nm) was supplied by Shanghai Runhe Nanotechnology Company in China. Cu_2_O@ZrP nanosheet was prepared in our group, and the detailed preparation process as well as its fabrication parameters are shown in Figures S1 and S2. Ethylenediaminetetraacetic acid disodium salt (EDTA-2Na), copper (II) sulfate pentahydrate (CuSO_4_·5H_2_O), ascorbic acid (AA), sodium hydroxide (NaOH) and other chemical reagents used in our experiment were commercial products purchased from Sinopharm Chemical Reagent CO., Ltd. *Escherichia coli* (*E. coli*, ATCC 25922), *Staphylococcus aureus* (*S. aureus*, ATCC 6538) and *Canidia Albicans* (*C. albicans*, ATCC10231) were provided by Guangdong Institute of Microbiology (Guangzhou, China). Other chemicals were used without further purification unless otherwise specified.

### 2.2. Preparation of PET/Cu_2_O@ZrP-B Fibers 

The PET resin was dried at 90 °C for 2 h under vacuum, and homogeneously mixed with a serious different content of nano-Cu_2_O@ZrP (0.1 wt %, 0.2 wt %, 0.4 wt %, and 0.6 wt % respectively) in a twin-screw extruder under conditions of 272 °C and 150 rpm (SHL-35, Shanghai chemical machinery co., LTD, Shanghai, China). Afterwards, the PET/Cu_2_O@ZrP-B hybrid fibers were prepared by using a melt spinning machine (Ube, Japan). The melt spinning conditions used were 282–293 °C and 800 m/min.

### 2.3. Preparation of PET/Cu2O@ZrP-I Fibers 

Firstly, a certain amount of nano-Cu_2_O@ZrP was dispersed in ethylene glycol (EG) under vigorous stirring for 2 h, and a following ultrasonication for 2 h under an ice water bath, forming a highly dispersed nano-Cu_2_O@ZrP suspension. At the same time, 2400 g of purified terephthalic acid (PTA) and 1200 g of Cu_2_O@ZrP/EG were added into a polymerization reactor equipped with a mechanical stirrer, and 1.2 g of ethylene glycol antimony was added acting as the polycondensation catalyst. The mixture was heated to about 180 °C under a flowing N_2_ atmosphere, whereupon water was generated. The esterification step finished after a theoretical amount of water was removed. The mixture was then heated up to 280 °C, meanwhile a vacuum (<50 Pa) was applied. The polycondensation was finished once the mechanical stir reached the assigned torque, which may have been done within 2–3 h. A series of PET/Cu_2_O@ZrP hybrid resin with different filler contents were prepared with the same method. Then, the PET/Cu_2_O@ZrP-I hybrid fibers were prepared by the same method of PET/Cu_2_O@ZrP-B hybrid fibers.

### 2.4. Antibacterial Activity of PET/Cu_2_O@ZrP Fibers 

Long-term antibacterial analysis: 5 g PET/Cu_2_O@ZrP-I-0.6% hybrid fibers was placed in 200 mL ultra-pure water under slow stirring with a magnetic stirrer at 30 and 80 °C. Then 2 mL solution was extracted through a 200 nm microporous filter membrane and sampled three times. Finally, the concentration of Cu in the solutions were measured by an inductively coupled plasma optical emission spectrometer (ICP-AES, Leeman Prodigy, DV, USA).

Antimicrobial assessment (shake-flask method): The strains were cultured in nutrient broth (0.5% peptone, 0.1% beef extract, 0.2% yeast extract, 0.5% NaCl, pH = 7.2) in an incubator overnight at 37 ± 1 °C. 7.5 g PET/Cu_2_O@ZrP hybrid fabrics were put into 100 mL of nutrient broth containing about 10^4^–10^6^ CFU/mL of *E. coli*, *S. aureus* or *C. albicans* and then shaken at 37 ± 1 °C for 24 h. 1.0 mL of the suspension was taken out from the test flask and diluted to a certain volume (to ensure the bacterial colonies grown could be counted easily and correctly) by a ten-fold dilution. The diluted solution was plated on Luria Bertani broth agar plate in triplicate and incubated at 37 ± 1 °C for 24 h. The number of bacterial colonies on each plate was counted. The killing rate (R) was relative to the viable bacteria counts as follows: $R=\frac{\left(Y-X\right)}{Y}\times 100\%$, where *Y* and *X* corresponded to the number of colonies incubated with the control and PET/Cu_2_O@ZrP hybrid fabrics, respectively.

### 2.5. Characterization 

Scanning Electron Microscopy (SEM, SU8010, Hitachi, Tokyo, Japan) was used to analyze the dispersity of Cu_2_O@ZrP in PET fibers. The X-ray diffraction (XRD) data were obtained at room temperature by a Japan Rigaku D/max-2550 PC X-ray diffractometer equipped with a Cu-Ka source (λ = 1.5404 Å) at a scanning rate of 10.0°/min, using a voltage of 36 kV and a current of 20 mA. Transmission electron microscopy (TEM) images were observed by using a JEOL JEM-2100F transmission electron microscope with an acceleration voltage of 200 kV. GPC-50 (Agilent, Palo Alto, CA, USA) was used to test the molecular weight and molecular weight distribution of PET and PET/Cu_2_O@ZrP hybrid resin, with hexafluoroisopropanol as the mobile phase with a flow rate of 1 mL/min. The breaking strength and elongation of the fibers were measured by the multifilament yarn strength tester (XL-1A, Shanghai New Fiber Instrument Co., Ltd., Shanghai, China) according to the GB/T3923.1-1997.

## 3. Results and Discussion

### 3.1. The Morphology of Nano-Cu_2_O@ZrP 

**Figure 1:**
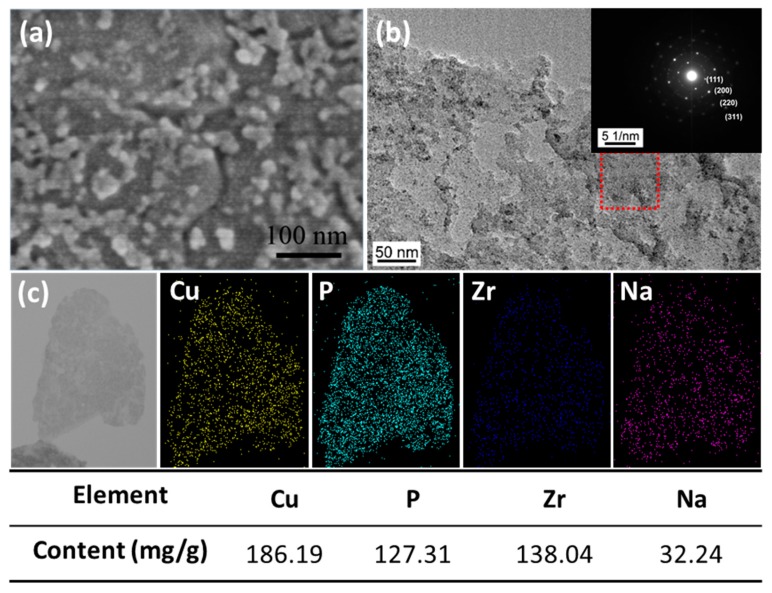
The structure analysis of nano-Cu_2_O@ZrP: (**a**) SEM image and (**b**) TEM image of Cu_2_O@ZrP. (**c**) Elemental mapping of the Cu_2_O@ZrP. Insert (b) shows selected area electron diffraction (SAED) patterns of the red square region. The data in the table is the element contents of Cu_2_O@ZrP.

The morphology of α-ZrP and nano-Cu_2_O@ZrP particles were thoroughly analyzed by TEM and SEM. SEM and TEM images (Figure S1) showed that the as-obtained α-ZrP nanosheet presented a smooth 2D nanostructure with a size of ~500–1200 nm. In contrast, after loading with Cu_2_O nanoparticles (Figure 1a), many small nanoparticles immobilized onto the surface of 2D α-ZrP nanosheet, which could be clearly observed. A high-resolution TEM image (Figure 1b) of a typical nano-Cu_2_O/ZrP particle and its corresponding selected-area electron diffraction (EDX) pattern (inset in Figure 1b) further indicated the uniform loading of small Cu_2_O nanoparticles (~10 nm) onto the surface of α-ZrP, showing an obvious and typical diffraction pattern of Cu_2_O crystalline [26,27]. Meanwhile, elemental mapping by energy dispersive X-ray spectroscopy (EDS) of the nano-Cu_2_O@ZrP further confirmed that a large amount of Cu_2_O nanoparticles had been successfully fabricated and evenly deposited onto the surface of ZrP nanosheets (Figure 1c). 

### 3.2. The Morphology of PET/Cu_2_O@ZrP Fibers

**Figure 2:**
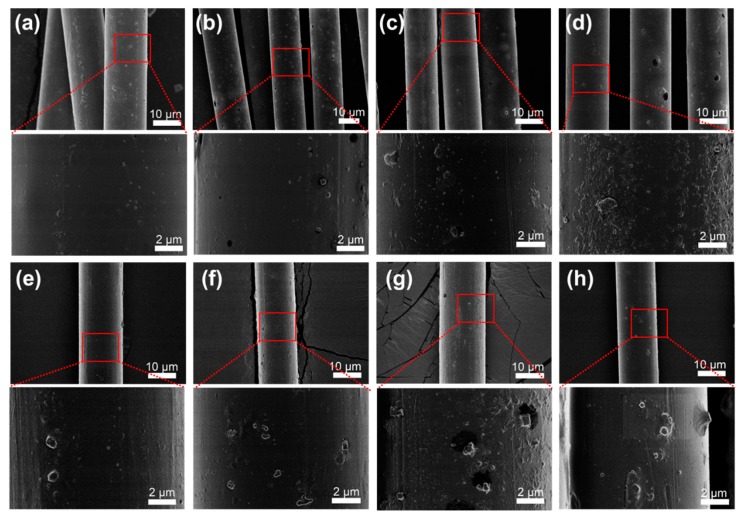
SEM images of (**a**) PET/Cu_2_O@ZrP-I-0.1%, (**b**) PET/Cu_2_O@ZrP-I-0.2%, (**c**) PET/Cu_2_O@ZrP-I-0.4%, (**d**) PET/Cu_2_O@ZrP-I-0.6%; (**e**) PET/Cu_2_O@ZrP-B-0.1%, (**f**) PET/Cu_2_O@ZrP-B-0.2%, (**g**) PET/Cu_2_O@ZrP-B-0.4% and (**h**) PET/Cu_2_O@ZrP-B-0.6%, respectively.

Figure 2 shows the SEM images of the in situ polymerized PET/Cu_2_O@ZrP (denoted as PET/Cu_2_O@ZrP-I) fibers in comparison with the neat PET fibers, and PET/Cu_2_O@ZrP fibers with the melt-blending method (denoted as PET/Cu_2_O@ZrP-B). As shown in Figure S3, neat PET fibers fabricated by a melt-spinning process only displayed a smooth surface. In contrast, after introducing nano-Cu_2_O@ZrP to PET fibers via either the in situ polymerization method or physical blending, both resulting PET/Cu_2_O@ZrP fibers demonstrated the ragged surfaces immobilized with many nanoparticles; and by increasing the adding amount of nano-Cu_2_O in the PET fibers matrix, the surficial particle number increased with their sizes growing larger and larger, demonstrating a growing aggregation of nano-Cu_2_O@ZrP [28]. Compared in detail, the particle size on the surface of the PET/Cu_2_O@ZrP-I fibers was significantly smaller than PET/Cu_2_O@ZrP-B fibers, which revealed that there should be a much better dispersibility of nano-Cu_2_O@ZrP in PET/Cu_2_O@ZrP-I fibers.

**Figure 3:**
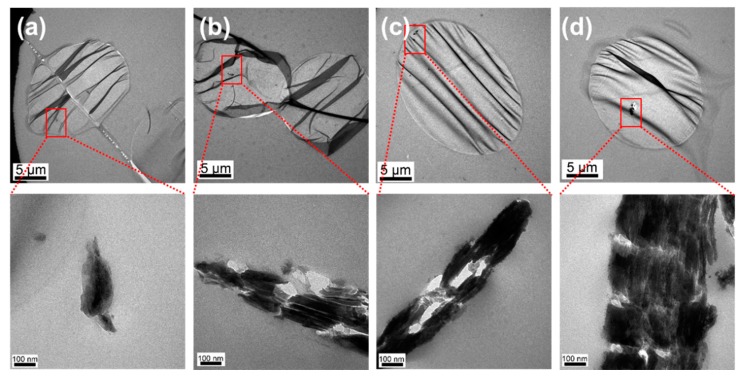
TEM images of (**a**) PET/Cu_2_O@ZrP-I-0.2%, (**b**) PET/Cu_2_O@ZrP-I-0.6%, (**c**) PET/Cu_2_O@ZrP-B-0.2% and (**d**) PET/Cu_2_O@ZrP-B-0.6%, respectively.

To further illustrate the dispersity of nano-Cu_2_O@ZrP inside the PET fibers matrix, cross-sectional TEM observation was carried out for both fibers of PET/Cu_2_O@ZrP-I and PET/Cu_2_O@ZrP-B. As shown in Figure 3, in both PET/Cu_2_O@ZrP fibers, nano-Cu_2_O@ZrP particle number increased by increasing the adding amount of nano-Cu_2_O@ZrP; additionally, it was clearly shown that the aggregation effect of nano-Cu_2_O@ZrP in PET/Cu_2_O@ZrP-I fibers (Figure 3a,b) was significantly lower than that in PET/Cu_2_O@ZrP-B fibers (Figure 3c,d), further demonstrating that the in situ polymerization process offers a much better dispersity of nano-Cu_2_O@ZrP in its resulting fibers. Moreover, as shown in Figure S3 and Table S1, it was clear that introducing nano-Cu_2_O@ZrP into the PET/Cu_2_O@ZrP fibers showed no apparent influence on their molecular weight as well as their molecular weight distribution of PET. The high magnification TEM image (Figure 3) showed a closer observation of nano-Cu_2_O@ZrP in PET fibers, which displayed an obvious 2D lamellar structure of the ZrP nanosheet immobilized with many small Cu_2_O nanoparticles. Such results further demonstrate that neither melt-blending nor in situ polymerization processes would not destroy the nano/micro structure of nano-Cu_2_O@ZrP hybrids [29].

### 3.3. The Effect of Introducing Nano-Cu_2_O@ZrP on Mechanical Properties and Crystallinity of the Resulting PET Fibers

**Figure 4:**
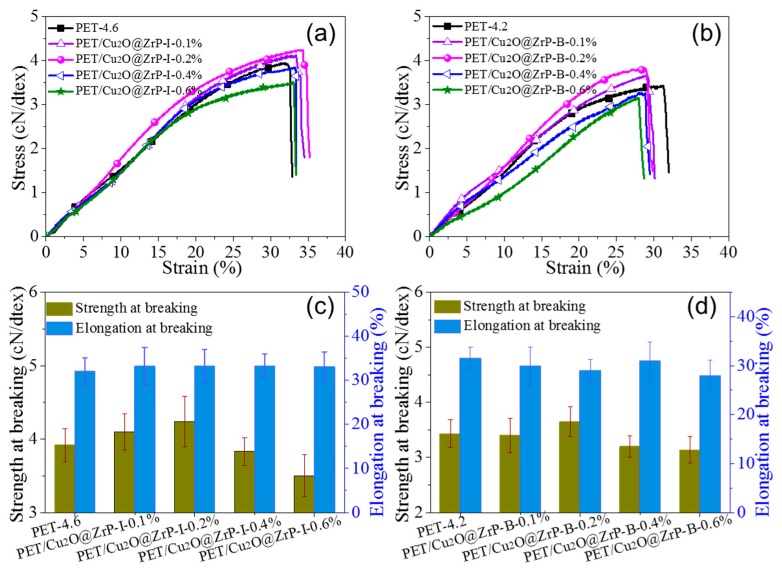
(**a**,**b**) Stress-strain curves and (**c**,**d**) mechanical performance of neat PET, PET/Cu_2_O@ZrP-I and PET/Cu_2_O@ZrP-B fibers, respectively.

In fabricating functional fibers by various methods, mechanical properties are always considered as one of the important indicators for their practical applications, which are mainly influenced by various parameters, including molecular weight, crystallinity and degree of orientation etc. [30,31]. Figure S4 shows that there is only a tiny difference in terms of molecular weight and molecular weight distribution between PET/Cu_2_O@ZrP-I and PET/Cu_2_O@ZrP-B fibers, which can be somewhat ignored. Additionally, under the melt spinning process with the other fabrication parameters constant, we evaluated the mechanical properties of both PET/Cu_2_O@ZrP fibers by testing their maximum drawing performance. The result in Figure 4 showed that with the same content of nano-Cu_2_O@ZrP, PET/Cu_2_O@ZrP-I fibers revealed an improved maximum drawing performance with a draw ratio value of 4.6 times, while its value of the PET/Cu_2_O@ZrP-B fibers was only 4.2 times. Such an improved mechanical performance can be attributed to the superior dispersibility of nano-Cu_2_O@ZrP particles inside the in situ polymerized PET fibers featuring much lower structural defects, hence obtaining a better drawability. Interestingly, as the content of nano-Cu_2_O@ZrP increased, the mechanical properties of the fibers showed an increasing tendency at the beginning until reaching an optimal content of nano-Cu_2_O@ZrP, and then decreased. Taking PET/Cu_2_O@ZrP-I as an example, at a nano-Cu_2_O@ZrP addition amount of 0.2 wt %, its fibers strength reaches the value of 4.24 cN/dtex, which is 0.28 cN/dtex higher than neat PET fibers. Moreover, the mechanical strength of the PET/Cu_2_O@ZrP-I fibers was much better than the PET/Cu_2_O@ZrP-B fibers under the same content of nano-Cu_2_O@ZrP additives. Such an enhanced mechanical performance was also closely related to the dispersion of nano-additives inside the PET fibers matrix. The nanoparticle aggregation will inevitably lead to a decrease in mechanical properties. Such a result can be further evidenced by analyzing their crystalline change of PET in the resultant hybrid fibers [5,32].

**Figure 5:**
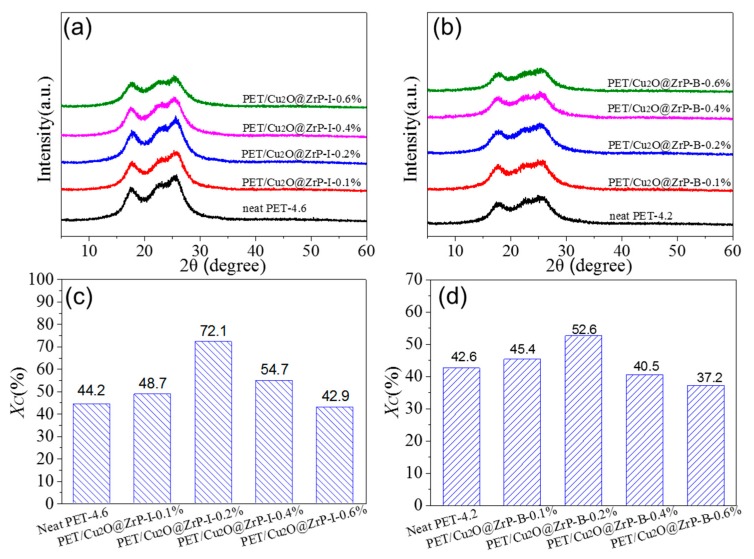
XRD curves of PET/Cu_2_O@ZrP-I and PET/Cu_2_O@ZrP-B (**a**,**b**) drawn fibers and (**c**,**d**) crystalline.

**Figure 6:**
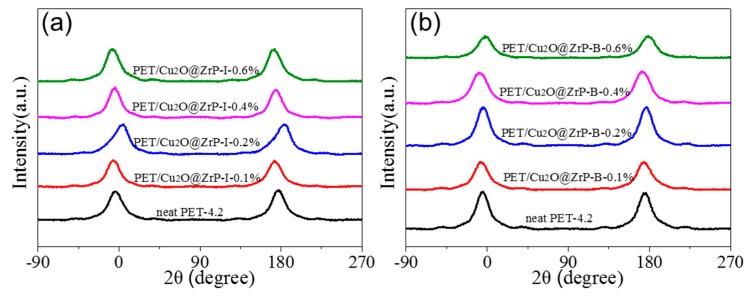
WAXD curves of (**a**) PET/Cu_2_O@ZrP-I and (**b**) PET/Cu_2_O@ZrP-B drawn fibers.

To further clarify the influence of nano-Cu_2_O@ZrP on the crystal structure of PET/Cu_2_O@ZrP hybrid fibers, the Wide-angle X-ray diffraction (WAXD) and orientation structure of both hybrid fibers were further investigated (Figure 5 and Figure 6). As shown in Figure 5, three obvious diffraction peaks could be observed at 17.7°, 22.8° and 26.3° for PET and its hybrid fibers, which were well assigned to the crystal plane [010], [110] and [100] of PET. 

The crystallinity of these fibers could be obtained by a fitting calculation and is listed in Figure 5c,d. With the content of nano-Cu_2_O@ZrP increasing, the crystallinity of PET also showed a trend of increasing first and then decreasing, which was consistent with the changing trend in their mechanical performance. These results were also consistent with their crystallinity calculated from the fibers enthalpy from the DSC test [33,34,35,36] (Figure S5, Tables S2 and S3). Such a phenomenon maybe due to the fact that, at a lower content, the nano-Cu_2_O@ZrP can be uniformly dispersed in PET matrix, which usually undergoes a heterogeneous nucleation process and hence induces an acceleration of crystallization; while excessive nano-Cu_2_O@ZrP particles will hinder the movement of the molecular chain [37]. To further discover the factors in improving mechanical properties of the PET/Cu_2_O@ZrP-I fibers, Figure 6 also shows the orientation curve of the fibers with the relevant calculated values recorded in Table 1. Similar to the fibers crystalline changing, with the increase of nano-Cu_2_O@ZrP content, the orientation of PET hybrid fiber showed an increasing trend first to a certain value and then decreased. A lower content of nano-Cu_2_O@ZrP induces the formation of uniformly distributed fine crystallites in the PET fibers matrix, which can then serve as a physical crosslinking point to improve the interaction between molecular chains. During the drawing process, the tensile stress can be uniformly transmitted leading to an orientation of the molecular segments along the axial direction of the fibers; anisotropic crystallites can be subsequently formed during the heat setting process improving the degree of crystal orientation of the fibers. However, once the content of nano-Cu_2_O@ZrP is higher than an optimal amount, the free movement of the molecular segment and crystallite at the interface of nano-Cu_2_O@ZrP will be restricted, the transmitted tensile stress cannot be separated from the Cu_2_O@ZrP bond; and the molecular segment and crystallite of the surface interface slip along the stretching direction, and the linear density of the fibers decreases, but the degree of orientation cannot be improved [38,39]. In comparison, the PET/Cu_2_O@ZrP-I hybrid fibers presents a higher orientation degree than PET/Cu_2_O@ZrP-B hybrid fiber, which is also mainly due to the good dispersibility of nano-Cu_2_O@ZrP in the PET polymer matrix. Therefore, under the same adding amount of nano-Cu_2_O@ZrP, PET/Cu_2_O@ZrP-I fibers present a higher fiber orientation and crystallinity, and offers the fibers better fracture strength with the value of 4.24 cN/dtex.

**Figure 7:**
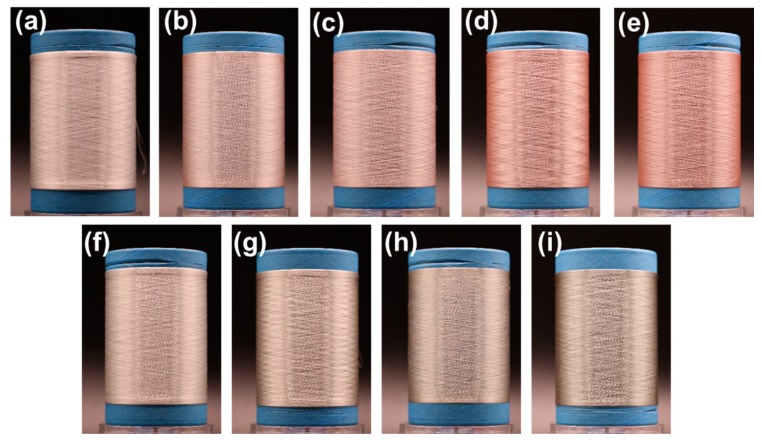
Digital photos of PET and PET-based fibers. (**a**) PET, (**b**) PET/Cu_2_O@ZrP-I-0.1%, (**c**) PET/Cu_2_O@ZrP-I-0.2%, (**d**) PET/Cu_2_O@ZrP-I-0.4%, (**e**) PET/Cu_2_O@ZrP-I-0.6%, (**f**) PET/Cu_2_O@ZrP-B-0.1%, (**g**) PET/Cu_2_O@ZrP-B-0.2%, (**h**) PET/Cu_2_O@ZrP-B-0.4%, and (**i**) PET/Cu_2_O@ZrP-B-0.6%, respectively.

At the same time, the ability to scale the fabrication of PET/Cu_2_O@ZrP hybrid fibers via such an in situ polymerization approach for engineering-level production has been further and unambiguously demonstrated. The digital photos of PET, PET/Cu_2_O@ZrP-I and PET/Cu_2_O@ZrP-B fibers are shown in Figure 7. It can be seen from Figure 7 that the PET/Cu_2_O@ZrP-I composite fibers exhibited the metallic luster of copper, while the PET/Cu_2_O@ZrP-B composite fibers exhibited a grayish green color. As the amount of nano-Cu_2_O@ZrP additive increased, the color of both PET/Cu_2_O@ZrP hybrid fibers gradually became darker. The PET/Cu_2_O@ZrP-B fibers showed a gray-green color with the main reason that partial oxidation of Cu_2_O had occurred under a high- temperature situation in the melt-blending-spinning processes.

### 3.4. Evaluation of Antibacterial Performance for the PET/Cu_2_O@ZrP Hybrid Fibers

**Figure 8:**
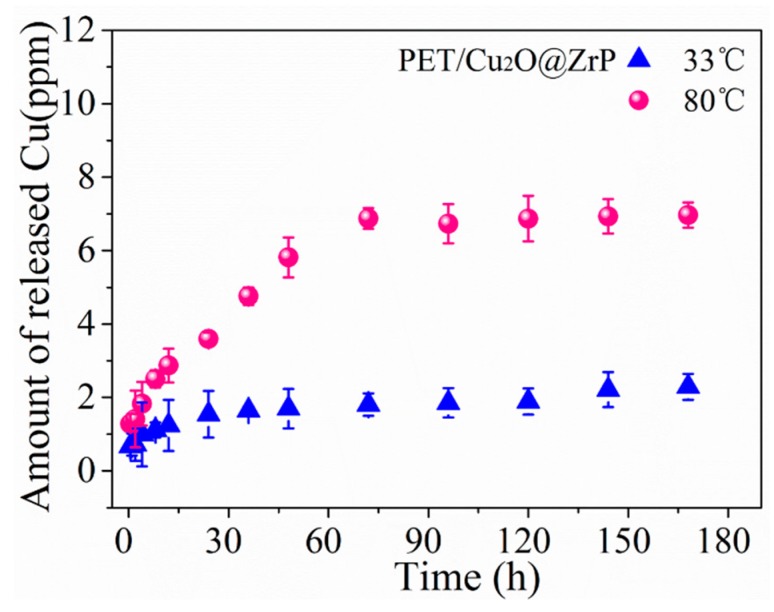
Cu release curves of PET/Cu_2_O@ZrP hybrid fibers on a daily basis in 200 mL ultrapure water at 33 °C and 80 °C.

Inside PET/Cu_2_O@ZrP-I hybrid fibers, nano-Cu_2_O@ZrP additives can achieve a good and stable dispersion, which can further induce the crystal structure of the fibers, thus improving its mechanical properties. Meanwhile, the antibacterial activities of Cu_2_O nanoparticles can maintain well on the surface of 2D ZrP as well as inside the resultant PET/Cu_2_O@ZrP-I fibers. In this article, we evaluated the antibacterial activity of our PET/Cu_2_O@ZrP-I fibers against three kinds of clinically isolated bacterium, i.e., *E. coli*, *S. aureus* and *C. albicans*. Before testing its antibacterial properties, we first evaluated the specific migration behavior of the PET/Cu_2_O@ZrP-I fibers in the biological environment [40,41], because it has been proven that the release of Cu highly depends on the moisture content and/or the temperature in a certain environment. In order to evaluate the Cu release in the PET/Cu_2_O@ZrP-I fibers, the release behavior was real-time monitored in 200 mL ultrapure water under two different temperatures, 33 °C and 80 °C respectively, which lasted for 7 days. As shown in Figure 8, a rapid release was shown during the beginning 3 days, and then the releasing reached a platform. The dissolution of Cu(I) due to oxygen can be illustrated by the following formula:

Cu(I) + O_2_ → Cu(II) + ·O^2−^

Meanwhile, higher Cu releasement was revealed under a high temperature (80 °C in this work). Even so, it was clear that only a small fraction (~14%) in the PET/Cu_2_O@ZrP fibers could release into the solution at a high temperature of 80 °C, revealing a good long-term action time. Conclusively, these results demonstrate that PET/Cu_2_O@ZrP-I fibers have different Cu release behaviors under different temperatures, which can meet different requirements of various applications.

To evaluate antimicrobial properties, three clinical isolated bacterial strains including gram-negative *E. coli* (ATCC 25922), gram-positive *S. aureus* (ATCC 6538), and fungus *C. albicans* (ATCC 10231) were selected for antibacterial tests, since they were closely related to practical medical-associated infections [41]. 

Table 2 showed the antibacterial performance of neat PET, PET/Cu_2_O, and PET/Cu_2_O@ZrP-I fibers after incubating with three types of bacterium for 24 h with their original bacteria concentration of about 10^4^–10^6^ CFU/ml [42,43]. As shown in Table 2, neat PET fibers showed no antibacterial activity in all the three types of bacterium. 

Additionally, the PET/Cu_2_O@ZrP-I fibers (i.e., PET/Cu_2_O@ZrP-I-0.2%) displayed a highly enhanced antibacterial activity against *C. albicans* in comparison with the PET/Cu_2_O fibers (i.e., PET/Cu_2_O-0.2%), although they presented similar antibacterial activities against both the *E. coli* and *S. aureus* bacterias. In specific, PET/Cu_2_O@ZrP-I fibers with a nano-Cu_2_O@ZrP content of 0.4 wt % (i.e., PET/Cu_2_O@ZrP-I-0.4%), displayed a 99% antibacterial activity against *C. albicans*; and its Cu content in Cu_2_O@ZrP was determined to be 186.2 mg/g as shown in Figure S2. In other words, only 0.07% Cu content in the PET/Cu_2_O@ZrP-I-0.4% could reach an excellent antibacterial performance (i.e., >99%). Furthermore, the antibacterial result in Table 2 demonstrates that PET/Cu_2_O@ZrP-I fibers present a superior antibacterial activity to the three selected types of bacteria, which is superior to both neat PET fibers and PET/Cu_2_O fibers.

## 4. Conclusions

In summary, based on an in situ polymerization method, we integrated antibacterial active nano-Cu_2_O@ZrP in a superior dispersion route into the PET matrix, and fabricated novel PET/Cu_2_O@ZrP-I antibacterial fibers with highly enhanced mechanical properties, when compared to the PET/Cu_2_O@ZrP-B fibers prepared by melt-blending. With such an approach, the nano-Cu_2_O@ZrP achieved superior dispersibility in the (obtained) PET/Cu_2_O@ZrP-I fibers confirmed by TEM and SEM studies, showing a higher PET crystalline than that of PET/Cu_2_O@ZrP-B fibers. Moreover, the chemical stability of nano-Cu_2_O as well as its antibacterial activities can be well-maintained in in situ polymerized PET/Cu_2_O@ZrP-I fibers by effectively preventing it from being oxidized during the fibers formation under a high temperature. Owing to the above merits, such a kind of PET/Cu_2_O@ZrP-I fibers reveals an efficient (>92%) and broad-spectrum antibacterial activity against *S. aureus*, *E. coli* and *C. albicans* even at a low nano-Cu_2_O@ZrP content of 0.2 wt %. More importantly, this work may open up a new pathway to uniformly integrate various functional nanomaterials into in situ polymerized materials (e.g., PET, PVA, PAN, etc.) for the fabrication of various advanced fibers.

## Figures and Tables

**Table 1:** Data of the full width at half maximum (*FWHM*) and preferential orientation (*Π*) based on Figure 5.

Samples	*FWHM (°)*	*Π (%)*	Samples	*FWHM (°)*	*Π (%)*
PET-4.6	17.8	95.4	PET-4.2	17.3	94.8
PET/Cu_2_O@ZrP-I-0.1%	17.7	94.6	PET/Cu_2_O@ZrP-B-0.1%	16.8	93.2
PET/Cu_2_O@ZrP-I-0.2%	18.9	92.8	PET/Cu_2_O@ZrP-B-0.2%	17.4	91.4
PET/Cu_2_O@ZrP-I-0.4%	19.3	91.6	PET/Cu_2_O@ZrP-B-0.4%	18.3	89.8
PET/Cu_2_O@ZrP-I-0.6%	21.8	90.9	PET/Cu_2_O@ZrP-B-0.6%	20.1	88.2

**Table 2:** The antibacterial activity of neat PET, PET/Cu_2_O, and PET/Cu_2_O@ZrP-I fibers respectively.

Bacterium	Samples	Blank Sample Viable Colonies (CFU/mL)	Viable Colonies (CFU/mL)	Microbial Reduction (%)
*E. coli*	PET	1.5 × 10^6^	1.5 × 10^6^	No effect
	PET/Cu_2_O-0.2%	1.5 × 10^6^	<1	>99
	PET/Cu_2_O@ZrP-I-0.2%	1.5 × 10^6^	<1	>99
*S. aureus*	PET	8.3 × 10^5^	8.3 × 10^5^	No effect
	PET/Cu_2_O-0.2%	8.3 × 10^5^	30	>99
	PET/Cu_2_O@ZrP-I-0.2%	8.3 × 10^5^	12	>99
*C. albicans*	PET	6.1 × 10^5^	6.1 × 10^5^	No effect
	PET/Cu_2_O-0.2%	6.1 × 10^5^	9.4 × 10^4^	85
	PET/Cu_2_O-0.6%	6.1 × 10^5^	1.9 × 10^4^	97
	PET/Cu_2_O@ZrP-I-0.2%	3.4 × 10^5^	2.8 × 10^4^	92
	PET/Cu_2_O@ZrP-I-0.4%	3.4 × 10^5^	35	>99

## Supplementary Materials

The following are available online at http://www.mdpi.com/2073-4360/11/1/113/s1, Figure S1: (**a**) SEM image and (**b**) TEM image of neat α-ZrP, Figure S2: (**a**) XRD patterns of ZrP and Cu_2_O@ZrP. (**b**) Cu 1p XPS spectra of Cu_2_O@ZrP, Figure S3: SEM images of neat PET fibers, Figure S4: GPC curve of (**a**) in-situ polymerization hybrid fibers and (**b**) blending hybrid fibers, Figure S5: DSC melting curves of PET/Cu_2_O@ZrP-I and PET/Cu_2_O@ZrP-B as-spun fibers (a, c) and drawn fibers (b, d), Table S1: PET/Cu_2_O@ZrP-I and PET/Cu_2_O@ZrP-B molecular weight and distribution, Table S2: PET/Cu_2_O@ZrP-I crystallinity of primary and drafting fibers, Table S3: PET/Cu_2_O@ZrP-B crystallinity of primary and drafting fibers.
