# Supplementary material for: Integrating Nano-Cu2O@ZrP into In Situ Polymerized Polyethylene Terephthalate (PET) Fibers with Enhanced Mechanical Properties and Antibacterial Activities
Source: Polymers (Basel). 2019 Jan 10;11(1):113. doi: 10.3390/polym11010113 (PMC6401950; doi:10.3390/polym11010113)
Supplement: Supplementary file 1 [file polymers-11-00113-s001.pdf]

Supplementary Materials:

Integrating Nano-Cu_2O@ZrP into In Situ Polymerized Polyethylene Terephthalate (PET) Fibers with Enhanced Mechanical Properties and Antibacterial Activities

Jialiang Zhou, Xiang Fei, Congqi Li, Senlong Yu, Zexu Hu, Hengxue Xiang *, Bin Sun and Meifang Zhu *

State Key Laboratory for Modification of Chemical Fibers and Polymer Materials, International Joint Laboratory for Advanced Fiber and Low-dimension Materials, College of Materials Science and Engineering, Donghua University, Shanghai 201620, China; zjl19871217@126.com (J.Z.); xiangfei@dhu.edu.cn (X.F.); licq1006@163.com (C.L.); 18317129897@163.com (S.Y.); wind007007@hotmail.com (Z.H.); sunbin@dhu.edu.cn (B.S.)

* Correspondence: hengxuexiang@dhu.edu.cn (H.X.); zhumf@dhu.edu.cn (M.Z.); Tel.: +86-21-6779−2849 (M. Z.)

Preparation of Cu_2O@ZrP naonosheet: Briefly, 10 g ZrP and 400 ml deionized water were mixed in a 500 ml three-neck flask under strong stirring and ultrasonic for 30 minutes at ambient temperature. 0.09 mol EDTA was slowly added to the suspension liquid and further stirred for 3 h at 40 °C. Then 0.15 mol $CuSO_4{\cdot}5H_2O$ was added to the complex solution with continued stirring for 30 min at 40 °C. Thereafter, 0.9 mol NaOH solution was injected into the mixture drop by drop. Next, 0.045 mol ascorbic acid solution was added to reduce the chelated cupric ions in situ. The hybrid material was separated by simple centrifugal separation, washed thoroughly with distilled water, and vacuum dryed at 60 °C before further characterization.

Characterization of Cu_2O@ZrP Nanosheet

The X-ray diffraction (XRD) data were obtained at room temperature by a Japan Rigaku D/max-2550 PC X-ray diffractometer equipped with a Cu-Ka source (λ=1.5404 Å) at a scanning rate of 10.0°/min, using a voltage of 36 kV and a current of 20 mA. UV-vis spectra were recorded by a Lambda 35 UV-Vis spectrophotometer. X-ray photoelectron spectroscopy (XPS) profiles were recorded and collected on an Axis Ultra DLD spectrometer (Thermo ESCALAB 250, USA) using a monochromatic Al Ka source. Transmission electron microscopy (TEM) images were observed by using a JEOL JEM-2100F transmission electron microscope with an acceleration voltage of 200 kV. Energy dispersive X-ray spectroscopy (EDS) experiments were carried out to analyze the composition of the samples.

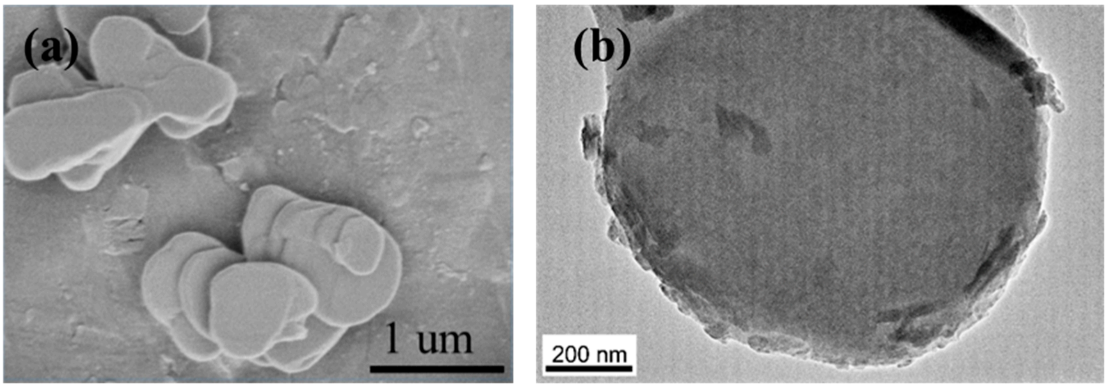

Figure S1. (**a**) SEM image and (**b**) TEM image of neat α-ZrP.

The SEM and TEM images of Cu_2O@ZrP are shown in Figure S1. It can be seen that the neat α-ZrP layer nanomaterial had a smooth surface and the particle size was between 500–1200 nm.

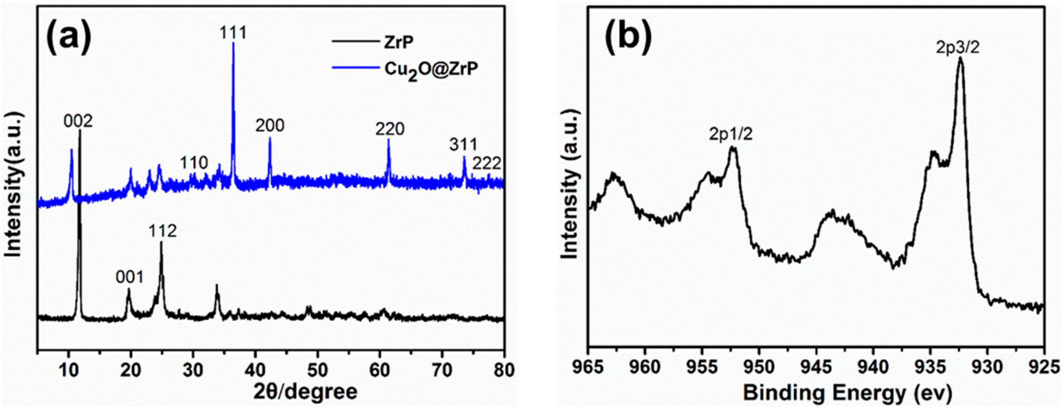

Figure S2. (**a**) XRD patterns of ZrP and Cu_2O@ZrP. (**b**) Cu 1p XPS spectra of Cu_2O@ZrP.

From the XRD curve of Figure S2, it can be seen that ZrP [002], [110], [112] grain surface diffraction peak corresponded to 2θ= 11.6, 19.8, 25.0, and the [110], [111], [200], [220] and [311] crystal surface of Cu_2O corresponded to 2θ= 29.51, 36.41, 42.31, 61.41 and 73.51 (JCPDS No. 05-0667). The characteristic diffraction peaks of $Cu(OH)_2$, CuO or Cu did not appear. By further testing the XPS curve of Cu_2O@ZrP, it was found that Cu 2p3/2 and Cu 2p1/2 were reflected in 932.5 eV and 952.6 eV peaks, further confirming the existence of Cu-O, which also indicated that the Cu_2O loaded onto the ZrP surface.

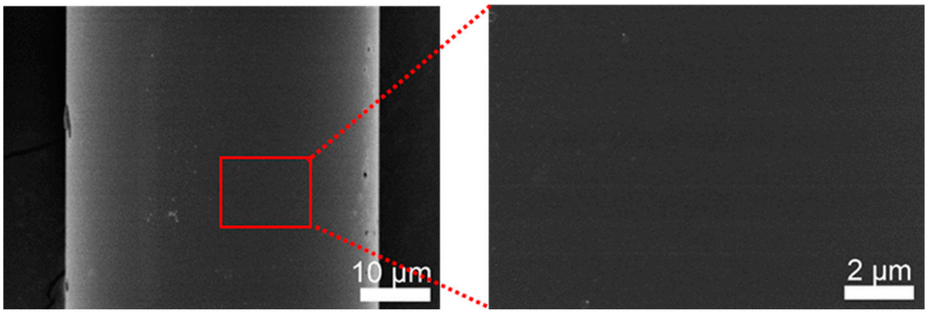

Figure S3. SEM images of neat PET fibers.

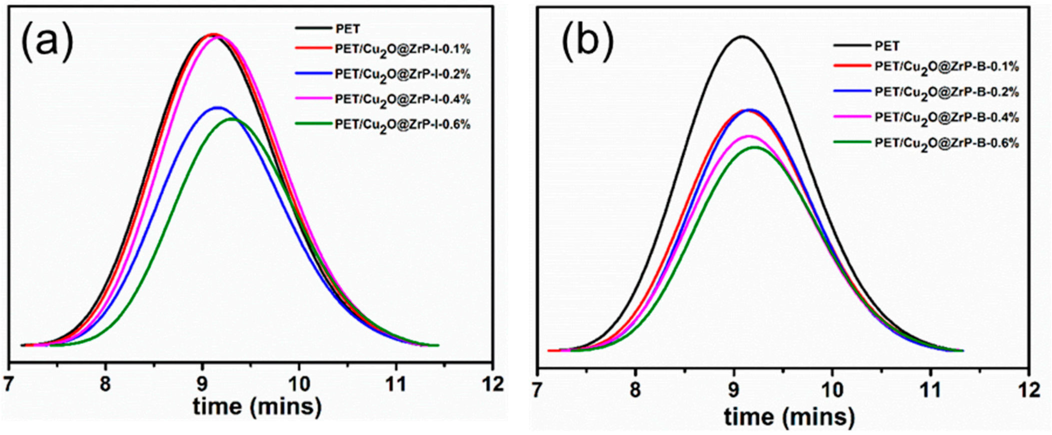

Figure S4. GPC curve of (**a**) in-situ polymerization hybrid fibers and (**b**) blending hybrid fibers.

Table S1. PET/Cu_2O@ZrP-I and PET/Cu_2O@ZrP-B molecular weight and distribution.

Samples	*Mn*	*Mw*	*PDI*	Samples	*Mn*	*Mw*	*PDI*
**PET**	15157	27094	1.7875	PET	15157	27094	1.7875
**PET/Cu_2O@ZrP-I-0.1%**	14474	25561	1.7301	PET/Cu_2O@ZrP-B-0.1%	13875	25044	1.7679
**PET/Cu_2O@ZrP-I-0.2%**	14039	24776	1.7388	PET/Cu_2O@ZrP-B-0.2%	13682	24530	1.7631
**PET/Cu_2O@ZrP-I-0.4%**	13769	24122	1.7648	PET/Cu_2O@ZrP-B-0.4%	12667	23344	1.7771
**PET/Cu_2O@ZrP-I-0.6%**	13362	23117	1.7659	PET/Cu_2O@ZrP-B-0.6%	11874	20409	1.7954

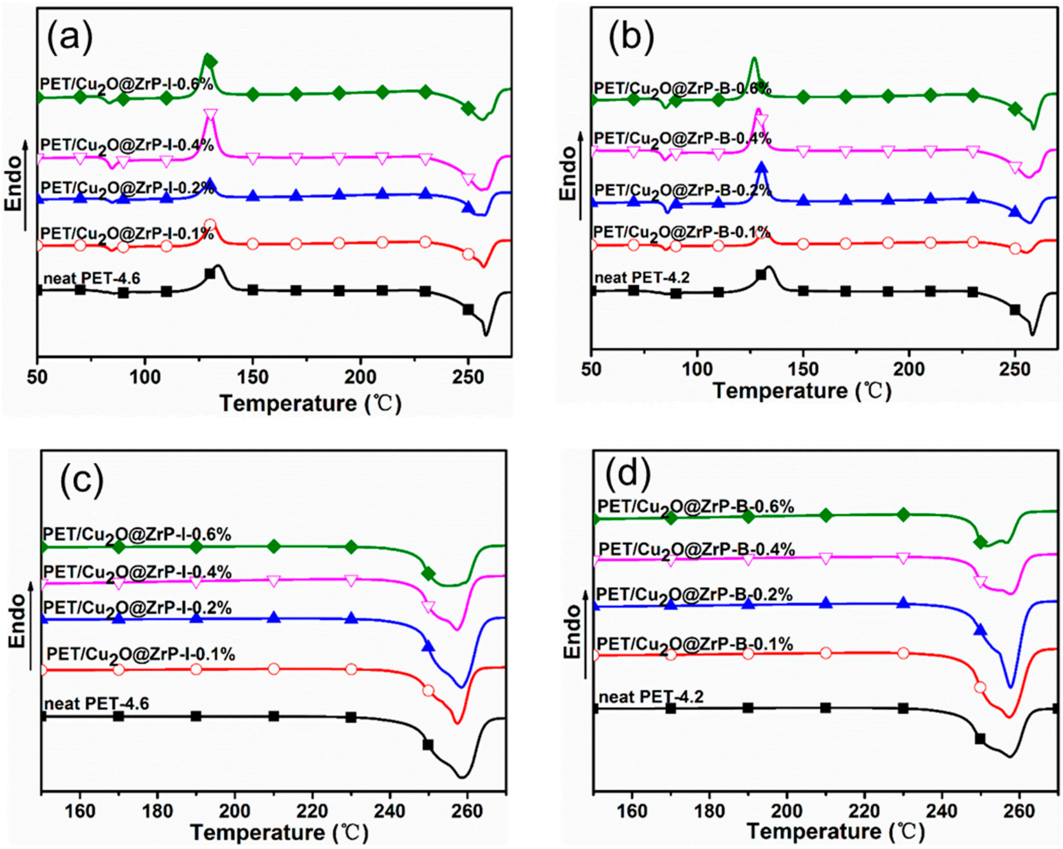

Figure S5. DSC melting curves of PET/Cu_2O@ZrP-I and PET/Cu_2O@ZrP-B as-spun fibers (**a**, **c**) and drawn fibers (**b**, **d**).

Figure S5 is the DSC curve of primary and drafting fibers of PET/Cu_2O@ZrP-I and PET/Cu_2O@ZrP-B. This was integrated to Figure S5 and the crystallinity of the hybrid fibers was calculated by the formula, and the column is listed in Table S2 and Table S3.

$$X_c = \frac{\Delta H_m - \Delta H_{cc}}{\varphi_i \Delta H_m^0}$$

ΔH_m: Melting enthalpy; ΔH_{cc}: Cold crystallization enthalpy when heating; φ_i: The mass fraction of the matrix.

Table S2. PET/Cu_2O@ZrP-I crystallinity of primary and drafting fibers.

Samples	As-spun filament		Drawn filament	
	ΔH(J/g)	*X_c(%)*	*ΔH(J/g)*	*X_c(%)*
PET	13.2	9.4	56.9	40.6
PET/Cu_2O@ZrP-I-0.1%	19.3	13.8	58.9	42.1
PET/Cu_2O@ZrP-I-0.2%	29.3	20.9	93.2	66.6
PET/Cu_2O@ZrP-I-0.4%	21.3	15.2	68.3	48.8
PET/Cu_2O@ZrP-I-0.6%	19.5	13.9	53.2	38

Table S3. PET/Cu_2O@ZrP-B crystallinity of primary and drafting fibers.

Samples	As-spun filament		Drawn filament	
	ΔH(J/g)	*X_c(%)*	*ΔH(J/g)*	*X_c(%)*
PET	13.2	9.4	54.3	38.8
PET/Cu_2O@ZrP-B-0.1%	13.5	9.6	55.4	39.6
PET/Cu_2O@ZrP-B-0.2%	20.6	14.7	56.6	40.4
PET/Cu_2O@ZrP-B-0.4%	19.0	13.6	52.7	37.6
PET/Cu_2O@ZrP-B-0.6%	17.6	12.6	46.4	33.1
